# Clinical Manifestations of Various Molecular Cytogenetic Variants of Eight Cases of “8p Inverted Duplication/Deletion Syndrome”

**DOI:** 10.3390/biomedicines10030567

**Published:** 2022-02-28

**Authors:** Darya A. Yurchenko, Marina E. Minzhenkova, Elena L. Dadali, Zhanna G. Markova, Galina E. Rudenskaya, Galina N. Matyushchenko, Ilya V. Kanivets, Nadezda V. Shilova

**Affiliations:** 1 Research Centre for Medical Genetics, 115522 Moscow, Russia; maramin@mail.ru (M.E.M.); genclinic@yandex.ru (E.L.D.); zhmark71@mail.ru (Z.G.M.); geruden@gmail.com (G.E.R.); matyushchenko@med-gen.ru (G.N.M.); nvsh05@mail.ru (N.V.S.); 2 Genomed Ltd., 115093 Moscow, Russia; dr.kanivets@genomed.ru

**Keywords:** inv dup del(8p), spacer, chromosomal/genomic imbalance, FISH, CMA

## Abstract

Inverted duplication syndrome with an adjacent terminal deletion of the short arm of chromosome 8—inv dup del(8p)—is a rare complex structural chromosomal rearrangement with a wide range of clinical manifestations. Molecular cytogenetic variants of chromosomal imbalance depend on the mechanism of rearrangement formation. We analyzed the clinical–genetic and molecular cytogenetic characteristics of the 8p inverted duplication/deletion syndrome, as well as the genotype–phenotype correlation in eight unrelated cases with the rearrangement of inv dup del(8p). The main clinical manifestations in all cases are psychomotor and language delay, muscle hypotonia, and dysmorphic facial features. Malformations of the central nervous system, such as corpus callosum agenesis, were found in five cases. Seizures were reported in only one case. We found that the cause of the formation of the rearrangement was generally ectopic recombination (seven out of eight cases) and this was due to U-type exchange in only one case. Depending on the mechanism of formation, the characteristics of the genomic imbalance were different, which made it possible to identify two molecular cytogenetic variants in the cases we describe here. No association between molecular cytogenetic variants and clinical manifestations was found.

## 1. Introduction

Inverted duplication with an adjacent terminal deletion of the short arm of chromosome 8—inv dup del(8p), ORPHA 96092—is a rare complex constitutional structural chromosomal rearrangement with an estimated frequency of 1/10,000–1/30,000 of newborns [1,2]. The first clinical case was described in 1976 by Weleber et al. They reported the birth of a patient with delayed psychomotor development and multiple developmental anomalies, including agenesis of the corpus callosum and cleft palate, and for the first time suggested a model for the formation of chromosomal imbalance [3].

To date, more than 100 postnatal cases with 8p inverted duplication/deletion syndrome have been published [4,5,6]. The clinical manifestations of this chromosomal/genomic imbalance include mild-to-severe intellectual disability, characteristic dysmorphic facial features, CNS malformations such as hypoplasia/agenesis of the corpus callosum (67%), cardiovascular diseases (65%), musculoskeletal problems (59%), hypotonia (88%), and seizures (55%). Dysmorphic facial features in individuals with inv dup del(8p) include microcephaly, large and prominent forehead, mildly arched eyebrows, deep-set eyes, ptosis or hooded eyelids, full cheeks, wide mouth, and micrognathia [4].

With the development of molecular and molecular cytogenetic research methods and the introduction of chromosomal microarray analysis into clinical practice, understanding of the mechanisms of formation of many chromosomal rearrangements, including inverted duplications/deletions, has changed [7,8]. Chromosomal/genomic imbalance in cases of inv dup del(8p) is seen as terminal deletion and inverted adjacent interstitial duplication that is localized in the short arm of one of the homologs of chromosome 8 [1]. In most cases, microarray analysis reveals part of a disomic region that is not involved in the imbalance—a spacer flanked by deletion and duplication regions of less than 5.5 Mb [1,9,10]. Such rearrangements are formed because of two consecutive events. The first (required) event is the formation of a symmetric dicentric chromosome in which breaks occur due to functional instability, and this leads to the formation of a monocentric chromosome with an inverted duplication having an adjacent deletion and a chromosome with a terminal deletion [11]. Dicentric chromosome 8 formation can occur by two different mechanisms: U-type exchange and non-allelic homologous recombination mediated by parental paracentric inversion inv(8)(p23.1) [4,12,13]. The latter mechanism is a consequence of ectopic recombination, which is most characteristic of inv dup del(8p); as a result of this mechanism, a chromosomal imbalance with an intervening disomic spacer is formed [1]. The formation of inverted 8p duplications/deletions due to U-type exchange occurs according to the “breakage–fusion–bridge” cycle mechanism that prevents the formation of the spacer [9,11,12,13]. Considering the characteristics of all previously described mechanisms of formation of inv dup del(8p), namely the presence or absence of a spacer and the size of duplication and deletion, it is obvious that different mechanisms of formation of chromosomal rearrangements lead to different molecular cytogenetic variants of inverted duplications/deletions of the short arm of chromosome 8. The literature contains little data on a clear connection between molecular cytogenetic variants of inv dup del(8p) and clinical manifestations in patients [1]. This report provides data on clinical–genetic and molecular cytogenetic characteristics as well as genotype–phenotype correlation in patients with 8p inverted duplication/deletion syndrome.

## 2. Materials and Methods

This study included eight unrelated cases (5 male, 3 female) aged 2 to 9 years. All cases are the only patient in a family. Their examination included genealogical analysis, neurological examination according to the standard technique, and MRI of the brain.

Chromosomal MicroArray analysis (CMA) was performed on DNA extracted from peripheral blood using an Affymetrix CytoScan HD array with 2.67 million probes, including 1.9 million copy number probes and 0.75 million SNP probes. Interpretation was made using the UCSC Genome Browser build hg19. The CytoScan HD array (Affymetrix, Santa Clara, CA, USA) was applied to detect the CNV across the entire genome following the manufacturer’s protocols. Microarray-based copy number analysis was performed using the Chromosome Analysis Suite software version 4.0 (Thermo Fisher Scientific Inc.: Waltham, MA, USA) and the results were presented on the International System for Human Cytogenomic Nomenclature 2020 (ISCN, 2020). Detected CNVs were totally assessed by comparing them with published literature and the public databases: Database of Genomic Variants (DGV) (http://dgv.tcag.ca/dgv/app/home, accessed on 10 January 2022), DECIPHER (http://decipher.sanger.ac.uk/, accessed on 10 January 2022) and OMIM (http://www.ncbi.nlm.nih.gov/omim, accessed on 10 January 2022). Genomic positions refer to the Human Genome February 2009 (GRCh37/hg19) Assembly (https://genome.ucsc.edu/;GRCh37/hg19assembly, accessed on 10 January 2022). The copy-number variants were evaluated according to ACMG recommendation [14].

Fluorescence in situ Hybridization (FISH) analysis was performed on chromosome spreads prepared from lymphocyte cultures. Multicolor banding (MCB) was carried out using the XCyte 8 mBAND probe (MetaSystems, Altlußheim, Germany). Subtelomeric DNA probes on chromosome 8 (Sub-Telomere 8pter; Sub-Telomere 8qter; Kreatech, The Netherlands) were used to confirm the deletion according to the manufacturer’s protocols. The data were analyzed using the ISIS software (MetaSystems, Germany) and the epifluorescence microscope AxioImager M.1 (Carl Zeiss, Oberkochen, Germany).

This study was approved by the ethical committee of the Research Centre for Medical Genetics (protocol No. 5/3 dated 12 November 2018). Voluntary informed consent was obtained from the legal guardians of the children considered in this scientific study. In all cases, informed consent was given to participate in the study and to publish the results.

## 3. Results

The clinical and genetic characteristics of the cases we observed are summarized in Table 1.

### 3.1. Clinical Findings

Prenatal and neonatal data are not presented, it is only known that low birth weight (3rd−10th centiles) was observed in four cases (Cases 1, 2, 4, and 7). All patients were characterized by a delay in early psychomotor and language development of varying degrees, craniofacial dysmorphisms, and hypotonia. The most common dysmorphic facial features were prominent forehead, upslanted palpebral fissures, thin upper lip, micrognathia, wide interdental spaces and low-set ears. Anomalies in the development of the central nervous system, such as agenesis/hypoplasia of the corpus callosum, were detected in MRI of five cases, Case 6 also had a pineal cyst, and Case 2 was the only case with a retrocerebellar cyst (Table 1).

Only Case 1 had epilepsy with afebrile focal seizures which first appeared at the age of 2 years and continued with varying frequency on modified anticonvulsant therapy; EEG video monitoring recorded discharges of epileptiform activity with a pre-dominance in left frontotemporal region. At the age of 7 years, he was seizure-free for 10 months, but epileptic activity on EEG persisted.

There were no cardiovascular diseases in the described cases.

### 3.2. Molecular Findings

**Figure 1:**
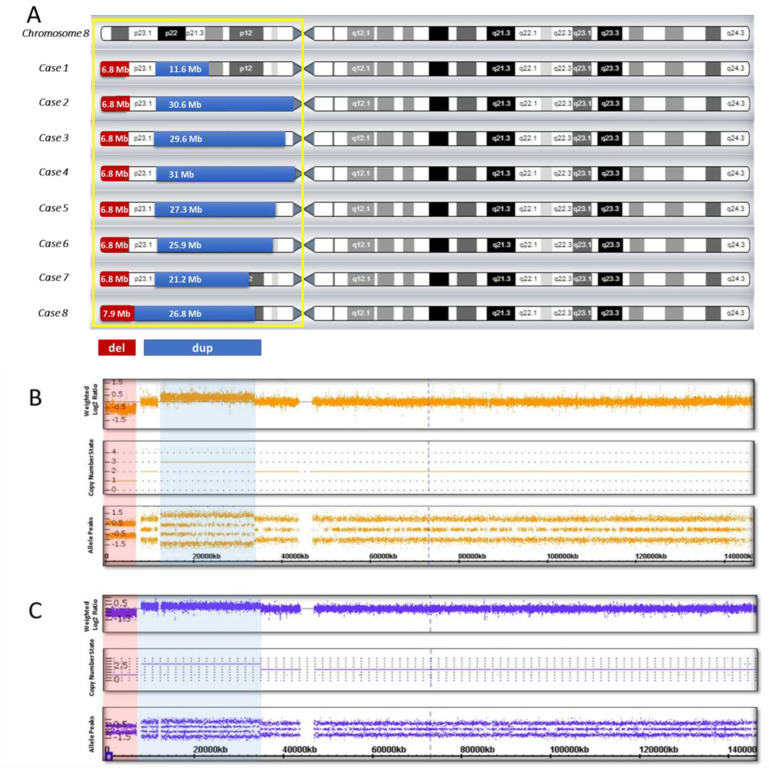
Genomic imbalance in eight cases with inv dup del (8p): (**A**) genomic pattern of the short arm of chromosome 8; (**B**) hybridization profile in the presence of a spacer (Case 7); (**C**) hybridization profile in the absence of a spacer (Case 8). The duplication region is shown in blue, the deletion region is shown in red.

CMA was performed for all eight cases (Table 1). Genomic imbalance in Cases 1 to 7 was seen as an approximately 6.8 Mb deletion, an intact region as a spacer in the range 4.9–5.5 Mb, and the sizes of the duplication in all our cases were different, from 11.6 to 31 Mb. In Case 8 the size of the deletion was 7.9 Mb, the size of duplication was 26.8 Mb, and the spacer was absent (Table 1, Figure 1).

**Figure 2:**
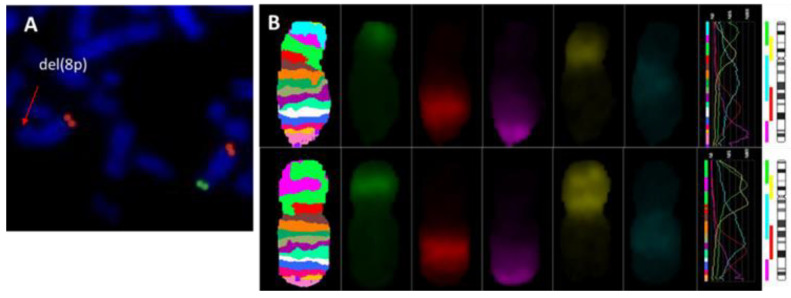
Metaphase FISH analysis. (**A**) FISH results with subtelomere probe for 8p(SpGreen)/8q(SpOrange); (**B**) MCB 8. Normal chromosome 8 banding pattern (**upper**), chromosome 8p with inv dup del (**lower**).

Subtelomeric FISH studies showed an 8p deletion in all cases (Figure 2A). Inverted orientation of the duplicated segment was confirmed in all cases (Figure 2B).

## 4. Discussion

An inverted duplication associated with a terminal deletion of the short arm of chromosome 8 is a rare chromosomal rearrangement, but it is one of the most frequent among all inverted duplications with an adjacent deletion [7,9,11]. This is due to the presence of two clusters of olfactory receptor genes or defensin repeats (ORDRs) localized in the 8p23.1 region—REPD (REPeat Distal) is a distal repeat and REPP (REPeat Proximal) is a proximal repeat. The region between the REPD and REPP, about 5 Mb in size, can be inverted, and it is considered a polymorphism [15]. It is important to note that the frequency of inversion heterozygotes is increased in the normal population (26% in the European population) [16]. It is believed that the presence of inversion polymorphism (inv(8)(p23.1)) in a heterozygous state in one of the parents, most often the mothers, is a predisposing factor to the formation of inv dup del(8p) through non-allelic homologous recombination [1,4,9,10].

Most cases of inv dup del(8p) reported in the literature are referred to as recurrent chromosomal rearrangements, implying ectopic recombination as the main mechanism of imbalance formation [1,4,9]. When an inverted 8p duplication/deletion is formed due to ectopic recombination, microarray analysis reveals a certain pattern of genomic imbalance, such as the obligatory presence of an interstitial duplication, an adjacent terminal deletion, and a disomic region—a spacer about 5–5.5 Mb in size [1,4,13]. In seven of our cases (Cases 1 to 7), we observed a similar picture in CMA: the size of the terminal deletion was similar in all the cases and was about 6.8 Mb, and there was a 5–5.5-Mb spacer, while the size of the duplication in each case was different (from 11.6 Mb to 31 Mb) (Table 1, Figure 1). For Case 8, the copy number variation (CNV) pattern was different, the size of the deletion was 7.9 Mb, the size of the duplication was 26.8 Mb, and there was no disomic region (Table 1, Figure 1). This picture (dup/del, absence of a spacer) is typical for non-recurrent inverted duplications/deletions formed due to U-type exchange [11,12,13].

Thus, here we describe eight cases of unrelated patients with 8p inverted duplication/deletion syndrome (5 males and 3 females aged 2 to 9 years), and their clinical and molecular cytogenetic data are presented in Table 1. Thus, based on the results of CMA, we can identify two molecular cytogenetic variants of inv dup del(8p) in our cases: the first variant is the presence of a recurrent deletion, a spacer, and a duplication of various size; the second is the presence of a non-recurrent deletion, a duplication, and the absence of a disomic region.

In our cases, both characteristic phenotypic features are present, and a degree of clinical variability was also observed (Table 1). The neurodevelopmental characteristics, such as intellectual disability, muscle hypotonia, and hypoplasia/agenesis of the corpus callosum, are frequent in individuals with inv dup del(8p) [4,5,6].

The region of chromosomal rearrangement inv dup del(8p) covers almost the entire short arm (~45 Mb) of chromosome 8 [4]. In their 2009 review, Tabare’s-Seisdedos and Rubenstein [17] reported that the 8p region contains 484 genes and 110 pseudogenes. However, for most of these a change in copy number, both in the form of haploinsufficiency and in the form of triplosensitivity, is not associated with the formation of defects and/or developmental anomalies [1,18].

Thus, the area of deletion (8p23.3–p23.1) in Cases 1 to 7 has almost the same size of about 6.8 Mb, and accordingly, a similar gene composition (Table 1, Figure 1). In Case 8, the deletion was more extended (7.9 Mb), but the beta-defensin gene clusters included in the deletion region is polymorphic in copy number among the individuals, and thus, it does not significantly contribute to the formation of the clinical manifestations in this case [18,19].

The 8p23.1→pter deletion region is considered a critical area associated with autism, speech delay, and epilepsy [20]. Several genes are distinguished in this region, whose influence may contribute to the formation of phenotypic manifestations: *DLGAP2* (OMIM 605,438), *CLN8* (OMIM 607,837), *ARHGEF10* (OMIM 608,236), *CSMD1* (OMIM 608,397), and *MCPH1* (OMIM 607,117) [20,21]. The *DLGAP2* gene is considered by many authors as a gene responsible for the development of progressive intellectual disability, and *CLN8* is thought responsible for intellectual disability and seizures [1,4,20,21,22,23]. The *ARHGEF10* and *CSMD1* genes are associated with development of the central nervous system [1,4]. The role of the *MCPH1* gene is discussed in the article by Ozgen et al., which described a patient with inv dup del(8p). Their Patient 1, a girl, had mild craniofacial dysmorphisms, autism spectrum disorder, and epilepsy, and the size of the deletion was 6.9 Mb. The researchers suggested an association of epilepsy and autism with loss-of-function *MCPH1* gene [21]. Interestingly, the contribution of the *CSMD1* and *MCPH1* genes to the formation of autism was also noted when these genes were involved in the duplication region [20,23]. In all our cases these genes entered the region of deletion, but only Case 1 had seizures, which prevents us from coming to a conclusion of the main contribution of the *CLN8* and/or *MCPH1* genes in the formation of epilepsy.

At the time of examination, autism spectrum disorders were not observed in our cases, probably due to early age (some of them up to five years), which makes a diagnosis difficult.

In all our cases there was a mental and language developmental delay, its degree varying from mild to severe (Table 1). Considering that all the cases except for Case 8 have almost the same size of the terminal 8p deletion, it is not possible to explain the reason for the varying severity of intellectual disability using deletion size data alone.

Anomalies of the cardiovascular system were noted in 65% of a large cohort of individuals with inv dup del (8p). [4]. None of our cases had congenital heart disease. Researchers C-P Chen et al. described a prenatal case of 8p inverted duplication/deletion syndrome in a fetus with multiple heart defects, and they suggested that its formation is associated with genes *SOX7* and *GATA4*, which are included in the deletion region [22]. Both these genes are involved in the duplication region in one of our cases, but no heart defect was observed (Case 8, Table 1). In other cases, when congenital heart disease was also not observed (Cases 1 to 7, Table 1), the copy number of genes *SOX7* and *GATA4* was not changed. Apparently, there is no evidence for triplosensitivity for these genes [18,22].

There are reports of a correlation between the size of a duplication and the severity of clinical manifestations in patients with inv dup del(8p), and it is the duplication that makes the main contribution to the heterogeneity of phenotypic manifestations [1,4,24]. In our cases, the size of the duplication varied from 11.6 Mb to 31 Mb, which allowed us to delineate the 11.6 Mb (12,490,998–24,107,465) minimal region of overlap among individuals with inv dup del(8p). The shared duplicated segment is consistent with the data of a large cohort (*n* = 49) of individuals with inv dup del(8p), in which the researchers calculated a similar region of 11–11.5 Mb [4]. In their article, Tabare’s-Seisdedos and Rubenstein [17] studied the gene composition of the short arm of chromosome 8 and correlated the dysfunction of genes localized in this region with clinical manifestations, such as impaired brain development, mental disorders, and diseases of the central nervous system (including epilepsy). Vibert R. et al. have proposed four candidate genes (*RHOBTB2*, *NEFL*, *CHRNA2*, and *GNRH1*) from the duplicated region that may result in a neurological phenotype in inv dup del(8p) patients [6]. Six cases we describe here are characterized by brain abnormalities, such as agenesis/hypoplasia of the corpus callosum, which was observed in five cases (Cases 3, 4, 6, 7, and 8). Case 6 was also characterized by a pineal cyst, and only Case 2 was diagnosed with a retrocerebellar cyst (Table 1). More than 40 genes on the short arm of chromosome 8 known to be involved in neuron development and function of the brain. Most of these genes are localized precisely in the duplication region in our cases and could make the main contribution to the formation of neurodevelopmental abnormalities (including malformations of corpus callosum) [17]. It is also important to note that In Case 1 with a duplication size of 11.6 Mb and in Case 5 with a 27.3 Mb duplication no brain developmental abnormalities were noted, while Cases 6, 7, and 8 (duplication size from 21.2 to 26 Mb) were characterized by agenesis/hypoplasia of the corpus callosum (Table 1). It is obvious that developmental abnormalities of the central nervous system do not correlate with the size of the duplications in the cases we studied.

Mental and language developmental delay as well as motor development of various degree were observed in all our cases (Table 1). However, we cannot relate the severity of mental and language developmental delay to the size of the duplication, since Case 1 with the smallest duplication size had a severe developmental delay, and Cases 2, 5, and 6 had mild-to-moderate delay.

Delayed motor development and hypotonia were observed in all our cases, but the severity of the manifestations varied. Case 1, whose duplication size was the smallest (11.6 Mb), had a slight delay in motor development and was characterized by hypotonia. In the remaining cases (Cases 2 to 8), the size of duplication was significantly more than 20 Mb, and all were characterized by a moderate-severe delay in motor development and hypotonia (Table 1). We note that the severity of delayed motor development correlates with the size of the duplication. In the publication of García-Santiago et al., hypotonia is explained by the involvement of the *NRG1* gene (OMIM 142445) in the duplication region (8p12), which plays an important role in the differentiation of the muscle spindle [1]. All our cases were characterized by hypotonia, but the *NRG1* gene entered the duplication region only in Cases 2 to 8. Considering that in Case 1 this gene is not included in the duplication region, it is obvious that it is not solely responsible for the imbalance which led to the formation of a clinical sign.

It is important to note that there is no reliable evidence that genes localized in the duplication region could have triplosensitivity [18]. Based on the results of our analysis of the phenotypic features and gene composition of the chromosomal region of imbalance, we did not come to an unambiguous conclusion about the main contribution of the duplicated overlap region, 11.6 Mb in size, for the clinical manifestations of inv dup del(8p) in our cases.

## 5. Conclusions

In summary, complex molecular cytogenetic studies using FISH and chromosomal microarray analysis allows to characterize 8p inverted duplication/deletion chromosome. Identification of the size and localization of genomic alterations enable to determine the mechanism of chromosomal rearrangement’s formation. All our patients had a similar deletion and different duplication. Our cases demonstrate core clinical features of developmental delay, craniofacial dysmorphism and a range of abnormalities on cerebral im-aging in patients with inverted duplication 8p syndrome, irrespectively of molecular cyto-genetic variants. There is no evidence support a correlation between duplication size and more severe clinical manifestations.

## Figures and Tables

**Table 1:** Clinical and molecular cytogenetic characteristics of eight cases with 8p inverted duplication/deletion syndrome.

Cases	Sex	Age (Years)	Mental and Language Development	Motor Development *	Craniofacial Dysmorphism	CNS Malformations on MRI	Coordinates ** of the Chromosomal Alterations and Size of the Rearrangement (CMA)
Case 1	M	7	Severe delay	Mild delay (unsupported walking since 18 months)	Sloping forehead, hypertelorism, upslanted palpebral fissures, wide nasal base, thin upper lip, micrognathia, malocclusion, large and deformed ears	No	arr[hg19] 8p23.3p23.1 (158048_6982980) x 1,8p23.1p21.2 (12490998_24107465) x 3 del: 6.8 Mb dup: 11.6 Mb spacer: 5.5 Mb
Case 2	M	2	Mild delay	Severe delay (not walking)	Hydrocephalic skull, large and prominent forehead, mild facial asymmetry, epicanthus, exophthalmos, strabismus, ptosis, wide nasal base, thin upper lip, mild micrognathia (more pronounced in infancy), wide interdental spaces, large and deformed low-set ears	Retrocerebellar cyst	arr[hg19] 8p23.3p23.1 (158048_6982980) x 1,8p23.1p11.1 (12527948_43169003) x 3 del: 6.8 Mb dup: 30.6 Mb spacer: 5.5 Mb
Case 3	F	9	Severe delay	Severe delay (unsupported walking since 7 years)	Epicanthus, wide nasal bridge, nares anteverted, abnormal growth of teeth, large dysplastic ears	Corpus callosum agenesis	arr[hg19] 8p23.3p23.1 (158048_6982257) x 1,8p23.1p11.21 (11936000_41509224) x 3 del: 6.8 Mb dup: 29.6 Mb spacer: 4.95 Mb
Case 4	M	5	Severe delay	Moderate delay (unsupported walking since 3 years 6 months)	Brachycephaly, large and prominent forehead, enophthalmos, mildly arched eyebrows, wide nasal base, nares anteverted, short philtrum, protruding lower lip, deformed low-set ears	Corpus callosum hypoplasia	arr[hg19] 8p23.3p23.1 (158048_6999114) x 1,8p23.1p11.1 (12592122_43673602) x 3 del: 6.8 Mb dup: 31 Mb spacer: 5.59 Mb
Case 5	F	3	Moderate delay	Moderate delay (walking with support since 2 years)	Moderate acrocephaly, flat occiput, wide interdental spaces, micrognathia, protruding low-set ears	No	arr[hg19] 8p23.3p23.1 (158048_6940661) x 1,8p23.1p11.22 (11935023_39246760) x 3 del: 6.8 Mb dup: 27.3 Mb spacer: 4.99 Mb
Case 6	M	2	Mild delay	Severe delay (not walking)	Prominent forehead, temporal balding, downslanted palpebral fissures, mildly arched eye-brows, wide nasal base, depressed nasal bridge, full cheeks, wide mouth, thin upper lip, micrognathia, low-set ears	Corpus callosum hypoplasia, pineal cyst	arr[hg19] 8p23.3p23.1 (158048_6944774) x 1,8p23.1p11.22 (12528482_38476919) x 3 del: 6.8 Mb dup: 25.95 Mb spacer: 5.58 Mb
Case 7	F	6	Severe delay	Moderate delay (unsupported walking since 4 years)	Narrow forehead, low anterior and posterior hairlines, upslanted palpebral fissures, mildly arched eye-brows, wide nasal base, nares anteverted, short philtrum, full cheeks, short chin, dysplastic ears	Corpus callosum hypoplasia	arr[hg19] 8p23.3p23.1 (158048_6982257) x 1,8p23.1p12 (12528482_33760127) x 3 del: 6.8 Mb dup: 21 Mb spacer: 5.5 Mb
Case 8	M	5	Severe delay	Moderate delay (unsupported walking since 2 years 3 months)	Prominent forehead, hypertelorism, upslanted palpebral fissures, thin upper lip, wide interdental spaces, large low-set ears	Corpus callosum agenesis	arr[hg19] 8p23.3p23.1 (158048_8093169) x 1,8p23.1p12 (8093169_34866530) x 3 del: 7.9 Mb dup: 26.8 Mb spacer is missing

* All cases are positive for hypotonia. ** All genomic positions are based on UCSC Genome Browser on Human Feb. 2009 (GRCh37/hg19) Assembly (https://genome.ucsc.edu/, (accessed on 10 January 2022); GRCh37/hg19 assembly).

## Data Availability

The data that support the findings of this study are available from the corresponding author upon reasonable request.
